# Genome-wide haplotype-based association analysis of key traits of plant lodging and architecture of maize identifies major determinants for leaf angle: *hap*LA4

**DOI:** 10.1371/journal.pone.0212925

**Published:** 2019-03-06

**Authors:** Carlos Maldonado, Freddy Mora, Carlos A. Scapim, Marlon Coan

**Affiliations:** 1 Institute of Biological Sciences, University of Talca, Talca, Chile; 2 Universidade Estadual de Maringá, Departamento de Agronomia, Maringá, PR, Brazil; Huazhong University of Science and Technology, CHINA

## Abstract

Traits related to plant lodging and architecture are important determinants of plant productivity in intensive maize cultivation systems. Motivated by the identification of genomic associations with the leaf angle, plant height (PH), ear height (EH) and the EH/PH ratio, we characterized approximately 7,800 haplotypes from a set of high-quality single nucleotide polymorphisms (SNPs), in an association panel consisting of tropical maize inbred lines. The proportion of the phenotypic variations explained by the individual SNPs varied between 7%, for the SNP S1_285330124 (located on chromosome 9 and associated with the EH/PH ratio), and 22%, for the SNP S1_317085830 (located on chromosome 6 and associated with the leaf angle). A total of 40 haplotype blocks were significantly associated with the traits of interest, explaining up to 29% of the phenotypic variation for the leaf angle, corresponding to the haplotype *hap*LA4.04, which was stable over two growing seasons. Overall, the associations for PH, EH and the EH/PH ratio were environment-specific, which was confirmed by performing a model comparison analysis using the information criteria of Akaike and Schwarz. In addition, five stable haplotypes (83%) and 15 SNPs (75%) were identified for the leaf angle. Finally, approximately 62% of the associated haplotypes (25/40) did not contain SNPs detected in the association study using individual SNP markers. This result confirms the advantage of haplotype-based genome-wide association studies for examining genomic regions that control the determining traits for architecture and lodging in maize plants.

## Introduction

Maize (*Zea mays* L.), along with rice and wheat, is one of the most important agricultural crops worldwide [1] and has been used as a food source [2] and as a raw material for pharmaceutical and agroindustrial products [1, 3], because of its nutritional composition (72% starch, 10% protein and 4% fat), versatility and broad adaptability. Usually, maize breeding programs have focused on obtaining gains in grain yield [4, 5]. However, the selection of cultivars based on traits related to lodging and the architecture of the plants has allowed important genetic advances in various breeding programs [1, 6, 7, 8, 9, 10, 11, 12, 13]. In fact, traits such as plant height (PH), ear height (EH) and the EH/PH ratio (or PH/EH) have important effects on plant lodging in intensive maize cultivation systems [12]. In addition, leaf angle (LA), an important determinant for the plant architecture, has been significantly improved over recent decades to adapt to the current planting density requirements, which has increased maize production [9]. Therefore, a better understanding of the genetic architecture of these traits would help during the processes of selecting and/or developing highly productive cultivars (or lines or hybrids).

The identification of genomic regions associated with quantitative traits, which are controlled by a large number of genes [1, 10, 11, 12, 14, 15, 16] and are greatly subject to environmental influence [17], has gained relative importance in the development of maize hybrids based on marker-assisted selection (MAS). In addition, in many maize populations, random events can cause "genotype x environment" interactions (e.g., the growing season), which can be difficult to interpret and quantify [18]. For this reason, the genetic stability of quantitative trait loci (QTL) must be verified over time (during different years or growing seasons) and/or under different environmental conditions [17, 19, 20].

Currently, the development of genotyping methods (via high-density arrays), such as genotyping by sequencing (GBS), has facilitated the identification of QTL for different traits of interest in various crops, including maize [1, 11, 21, 22], and the exploration of adjacent genes, using genome-wide association studies (GWAS) [22, 23, 24]. The use of single-nucleotide polymorphisms (SNPs) has proven to be a powerful tool in breeding programs for different agricultural crops [1, 21, 25, 26]. In this sense, the use of haplotypes, defined from SNP arrays with high linkage disequilibrium (LD), has emerged as a methodological variant in the identification of genomic regions and/or in the prospecting of candidate genes from GWAS. This analysis approach considers the natural dependence that exists between SNPs [27], which becomes more relevant when considering high-density DNA arrays.

A haplotype is defined as a set of nearby SNPs (polymorphic), with a strong linkage disequilibrium between them [22]. From this point of view, it is important to consider that some SNPs are in strong linkage disequilibrium [27], and therefore, the development of GWAS based on haplotype blocks may represent a more efficient alternative than the use of individual markers [28]. In addition, the use of haplotypes can compensate for the biallelic limitation of the SNPs, improve the efficiency of identifying QTLs [22], and provide knowledge regarding genetic determinants that cannot be captured by individual or independent marker approaches [29]. For example, Chen et al. [30] and Contreras-Soto et al. [22] detected haplotypes associated with resistance to maize ear rot [3] and different agronomic traits in soybeans (plant height, seed yield and weight of 100 seeds) [22]. Therefore, with the intention of compensating for the limitations of association analyses based on individual SNP markers [22], the following objectives were proposed in the present study: (i) determine haplotype blocks based on a set of high-density SNPs and identify haplotype-trait associations using GWAS for traits related to plant architecture and lodging (LA, PH, EH and the EH/PH ratio); (ii) compare the results of GWAS based on haplotypes with their counterparts based on individual SNP markers; (iii) determine the stability of the associations over two consecutive growing seasons; and (iv) examine the genomic regions flanking the genetic associations that were determined by GWAS.

## Materials and methods

### Description of the biological material and the phenotypic evaluation

A genome-wide association study (GWAS) was conducted on a panel containing 183 inbred lines of tropical maize, which were evaluated during the 2014–2015 and 2015–2016 growing seasons in the town of Iguatemi (latitude: 23S 25'; longitude: 51W 57' and altitude: 550 m), located in the experimental fields of the Department of Agronomy of the State University of Maringá (UEM), Brazilian state of Paraná. The lines were planted according to a randomized complete block design, with 2 blocks and 28 repetitions per line. The field management was performed using artificial irrigation and according to the agronomic practices suggested by Cruz et al. [31].

The following traits related to plant lodging were evaluated: total plant height (PH), ear height (EH), and the EH/PH ratio. PH was measured from the ground to the tip of the primary inflorescence. EH was measured from the ground to the node of the first ear. The EH/PH ratio was obtained by dividing EH by PH in each repetition. In turn, the leaf angle (LA), one of the primary determinants of plant architecture, was measured in each repetition 10 days after anthesis. Four leaves above the first ear were examined to visually estimate the LA, which was measured on a categorical scale with 4 levels: (1) an inclined angle, (2) erect, (3) very erect, and (4) extremely erect.

### Genomic DNA and SNP discovery

Genomic DNA was isolated from the leaf tissue of inbred lines, according to the protocol established by Coan et al. [21]. Subsequently, the DNA samples were sent to the Genomic Diversity Institute of Cornell University for SNP discovery via genotyping by sequencing (GBS), which is described in Elshire et al. [23] and at https://bitbucket.org/tasseladmin/tassel-5-source/wiki/Tassel5GBSv2Pipeline (Institute of Genomic Diversity, Cornell University). The description of the GBS method used can be summarized as follows: (I) the reduction of genome complexity by digestion with restriction enzymes sensitive to ApeKI methylation; (II) the ligation of adapters (compatible with restriction enzymes) to the genomic DNA fragments; (III) the grouping, purification and amplification of the genomic material by means of Illumina sequencing primers (with a binding site specific to the ligated adapters) and PCR; (IV) the purification of the PCR product and the confirmation of the fragment sizes within the GBS libraries by means of a DNA analyzer (BioAnalyzer, Agilent Technologies, Inc., USA); and (V) the quantification and dilution of the libraries for subsequent sequencing in an Illumina HiSeq 2000 (Illumina Inc., San Diego, CA).

Once the reading sequences were obtained (raw data), TASSEL 5.2 software [32] was used to align the data with the *Zea mays* version *AGPV3* reference genome (B73 RefGen v3) [33]. After the alignment, the heterozygosity and the variants of Minor Allele Frequency (MAF) were calculated using VCFtools (version v0.1.12a). The readings with a quality score below 80 on the Phred scale (genotyping quality) [34] were eliminated. Finally, the database was filtered considering an MAF > 0.01 and a proportion of missing data per site < 90% [35].

### Linkage disequilibrium (LD)

The LD analysis was performed in TASSEL 5.2 [32], using SNPs that had less than 25% missing data and an MAF > 0.05. The LD between marker pairs was estimated using the correlation coefficients of the allelic frequencies (*r*^*2*^) considering all the possible combinations of the alleles. The level of significance (*p*-value) was calculated using 10,000 permutations.

The critical *r*^*2*^ value was estimated according to Breseghello and Sorrells [36] and Laidò et al. [37], using the transformation of the square root of the *r*^*2*^ values and taking the 95th percentile of these data as a threshold for which the LD is likely to be caused by a real physical linkage. Finally, the LD patterns were evaluated by means of a non-linear regression between the *r*^*2*^ values and the physical distance of the SNPs (in bp) [38].

### Haplotype blocks

A haplotype-block is defined as arrays of two or more SNP with high linkage disequilibrium. Haplotype blocks were constructed for each chromosome, considering SNPs with a MAF greater than 0.05 and with less than 25% missing data, using HAPLOVIEW [39]. The formation of the blocks was performed using the confidence interval method, described by Gabriel et al. [40] and implemented in HAPLOVIEW. This method considers the 95% confidence intervals of the D’ values, classifying the LD as "strong LD", "inconclusive", or "strong recombination". Finally, blocks were built if 95% of the comparisons were of the "strong LD" type. Haplotype blocks were later transformed into multiallelic markers, for the subsequent haplotype-trait association analyses, regarding the allelic combinations within each block to be independent alleles.

### Population structure and estimation of the kinship matrix

The kinship matrix was calculated based on identity-by-state (IBS) [41], using TASSEL 5.2 [32]. The population genetic structure was inferred using a Bayesian clustering model, available in STRUCTURE 2.3.4 [42], with a subset of markers without missing data (~ 5,000 SNP). For the STRUCTURE program, twenty runs were considered for each possible K (number of populations) ranging from 2 to 10, with the admixture model [42], correlated allele frequencies, a burn-in period of 1x10^5^, and 1x10^6^ MCMC repetitions. The optimal value of K was estimated from the second-order change rate of the probability function with respect to K (*ΔK*), as proposed by Evanno et al. [43]. Additionally, a principle component analysis (PCA) was performed in TASSEL and dendrogram was constructed according to the hierarchical neighbor joining method using DARWIN 5.0.158 [44] to visualize and corroborate the results from STRUCTURE.

### Statistical analysis of the phenotypic data

The analysis of the phenotypic data was performed using the following mixed linear model, with SAS software (SAS Institute, Inc., Cary, NC):

$$y=X\beta +Z_{1}l+Z_{2}ls+\varepsilon$$

where **y** represents the phenotypic values (for LA, PH, EH or the EH/PH ratio); **X**, **Z**_**1**_ and **Z**_**2**_ correspond to the known incidence matrices that relate **y** with the vectors **β**, **l**, and **ls**, respectively; **β** is the fixed effect vector of the season and the nested block within each season; **l** corresponds to the vector of random genotypic effects (lines); **ls** is the random effects vector due to the line-season interaction; and **ε** is the vector of the random residual effect. The restricted maximum likelihood (REML) method was used for the estimation of variance components. The following information criteria were used to determine the statistical significance of the random effects (model comparisons):

$$\begin{array}{l} BIC=-2\;\log\;RL+d\log n\\ AIC=-2\;\log RL+2d \end{array}$$

where BIC corresponds to the Schwarz (Bayesian) information criterion; AIC is the Akaike information criterion; -2log *RL* denotes the maximum value of the log likelihood (restricted); *d* corresponds to the dimension of the model; and *n* is the number of valid observations for the estimation of RL. The best model was the one that minimized the value of the information criteria [45].

The variance components of all the traits were determined using ASREML 4 [46]. For leaf angle, the statistical analysis was performed using a generalized linear mixed model (GLMM) because it supports ordinal variables. GLMM uses an approximate probability technique called penalized quasi-likelihood (PQL) [47], also known as pseudo-likelihood [48], which is based on an approximation of the first-order Taylor series.

Heritability (*H*^*2*^) was calculated for all traits using the following expression:

$$H^{2}=\frac{\sigma_{g}^{2}}{\sigma_{g}^{2}+\left(\sigma_{gs}^{2}/n\right)+\left(\sigma_{\varepsilon }^{2}/n\cdot r\right)}$$

where $\sigma_{g}^{2}$ is the genotypic variance, $\sigma_{gs}^{2}$ represents the variance of the interaction between the genotype and the environment, $\sigma_{\varepsilon }^{2}$ corresponds to the residual variance, *n* is the number of environments (growing seasons), and *r* indicates the number of repetitions.

### Genome-wide association study (GWAS)

Both association analyses, haplotype-trait and SNP-trait, were performed using a mixed linear model (MLM) in TASSEL 3.0 and TASSEL 5.2, respectively. The statistical model considers the effects of the population structure (Q) and genetic relationships or matrix kinship (K) among inbred lines, which has the following expression:

y=Sα+Qv+Zμ+ε

where *y* is the vector of adjusted phenotypic observations (Adjusted Entry Means) [49], **α** is the vector of SNP molecular markers or haplotype blocks (fixed), **v** is the vector of the effect of the population structure (fixed), **μ** is the vector of polygenic effects or genetic background (random), and **ε** is the vector of residual effects. **S**, **Q** and **Z** are the incidence matrices of the associated vectors.

The SNP markers with a MAF less than 0.05 and with missing data greater than 25% were excluded from the association analyses to reduce the false positive rate (type I error) [50].

### Trait correlations and pleiotropic

Trait correlations were calculated according to Pearson’s correlation coefficient in the R-project [51] software. Additionally, the probability that a locus is truly associated with more than one trait was evaluated by the logarithm of Bayes Factor (log10 (BF)) and the Posterior Probability of Association (PPA) [52]. The PPA was calculated as follows:

$$\mathrm{PPA}=\frac{\left(\mathrm{BF}\times \pi \right)}{\left(1-\pi \right)+\left(\mathrm{BF}\times \pi \right)}$$

where π is the prior probability that a given SNP is truly associated with the trait of interest (π = 1.0 × 10–3). BF was calculated using a Bayesian multivariate regression analysis in the SNPTEST software [53].

### Reference genome

Genes and QTLs recorded in the maize genome *AGPV3* (B73 RefGen_v3), available in the *MaizeGDB* database (http://www.maizegdb.org//) [54], were used as references. For this, a window (or threshold) of twice the distance indicated by the LD (0.9 kb, plus the size of the haplotype) was established, placing the SNP or haplotype in the center of the window.

## Results

According to the information criteria, the model that best fits the PH, EH and EH/PH data was the complete model (M3), which includes the effect of line-season (LxS) interaction (Table 1), while for LA, the model without LxS interaction (M2) was the best fit model. Moreover, based on the results of AIC, it was determined that there were no differences between the M2 and M3 models (Δ*i*<2) [55] for LA. The moderate coefficients of variation (CV) found in the four traits (19% in LA, 10% in PH, 16% in EH and 11% in the EH/PH ratio) indicate an adequate level of experimental precision. The average values for PH (1.52 and 1.27, respectively) and EH (0.81 and 0.58, respectively) differed between growing seasons, with higher values observed during 2014–2015, indicating that the environment had an impact on variation for these traits. On the other hand, the LA trait did not show significant changes between seasons (1.287 and 1.291, for 2014–2015 and 2015–2016, respectively). The high values of heritability (*H*^*2*^ = 0.95, 0.74, 0.94 and 0.83, for PH, LA, EH and the EH/PH ratio, respectively) were similar to those reported in previous studies [1, 7, 15, 56]. The strong genetic control observed in this maize panel increases the detection power of genetic regions associated with the traits [57].

**Table 1:** Model selection results based on the Bayesian (BIC) and Akaike (AIC) information criteria for leaf angle (LA), ear height (EH), plant height (PH) and the EH/PH ratio in inbred maize lines. The model corresponds to a model that does not include the effects of line and the environment-line interaction; the model considers the effects due to line, and the model corresponds to the complete model.

Model	LA			EH			PH			EH/PH		
	-2LRL	AIC	BIC	-2LRL	AIC	BIC	-2LRL	AIC	BIC	-2LRL	AIC	BIC
M1	1025.0	1027.0	1031.4	-1109	-1107	1101	529.5	531.5	531.5	-5116	-5114	-5108
M2	324.0*	328.0*	334.0*	-2941	-2937	-2931	-1607	-1603	-1597	-5808	-5804	-5798
M3	324.2	328.2	334.2	-3247*	-3241*	-3232*	-1953*	-1947*	-1938*	-6578*	-6570*	-6558*

*Model of best fit; -2LRL denotes the maximum value of the log likelihood (restricted).

Correlations between the traits related to plant lodging were positive and statistically different from zero (p-value < 0.001), with values of *r* = 0.89 (EH and PH), *r* = 0.80 (EH and the EH/PH ratio), and *r* = 0.42 (PH and the EH/PH ratio). On the other hand, LA showed a low negative correlation (*r* varying from -0.17 to -0.24) with traits related to plant lodging. The high correlation between EH and PH indicates that as plant height increases, so does the ear height, avoiding an increase in EH/PH ratio. On the other hand, the EH/PH ratio presented a better correlation with EH than with PH, which could indicate that this trait is mainly influenced by a change in EH.

### Population structure

**Fig 1:**
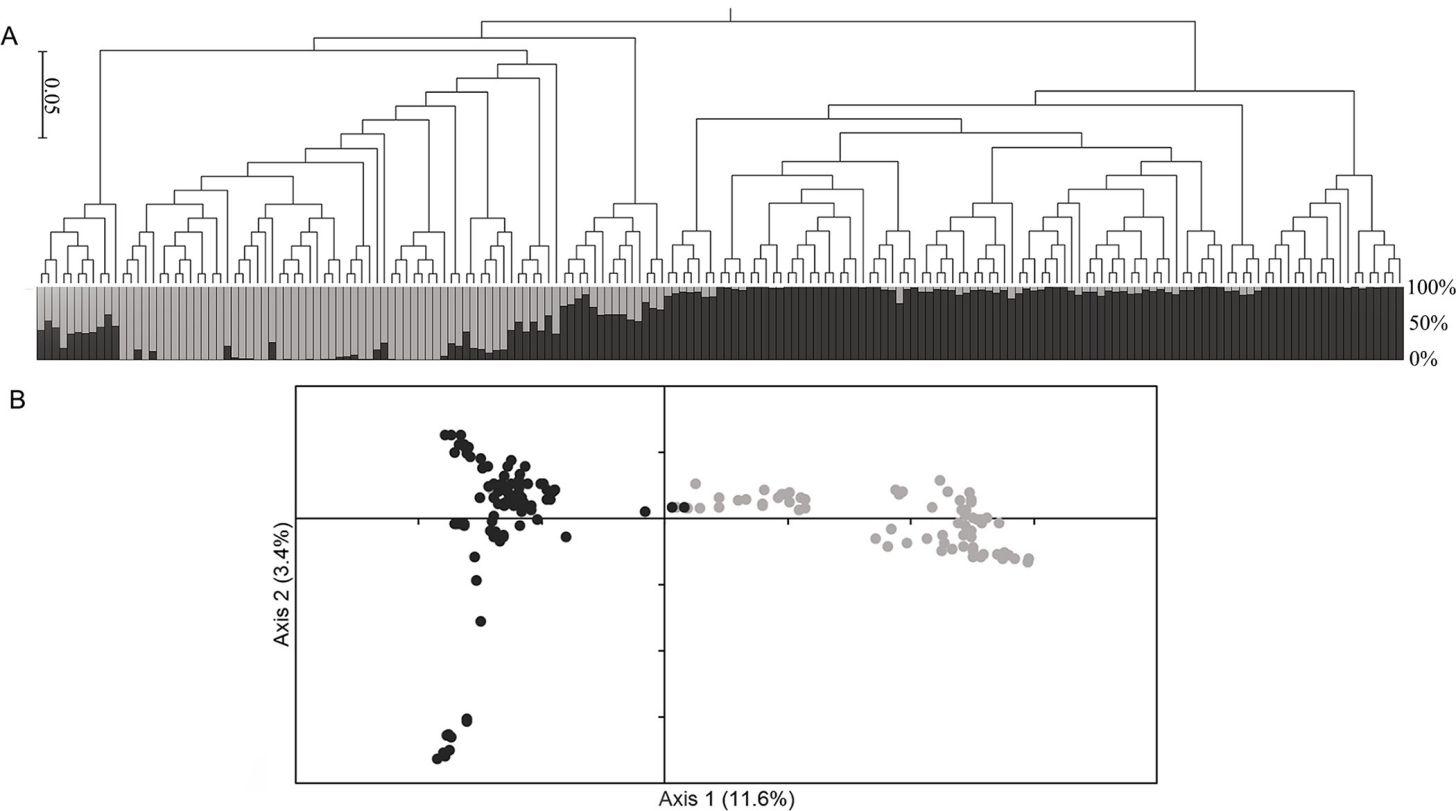
Distribution of the 183 inbred maize lines in the two sub-populations (field corn and popcorn) using a subgroup of 5000 SNP markers distributed on all chromosomes. (A) Genetic structure inferred by a Bayesian clustering model using STRUCTURE and a dendrogram obtained using the neighbor joining based on Nei's genetic distances. The proportion of colored segments light gray and dark gray indicates the proportion of the genome extracted from the two sub-groups (field corn and popcorn, respectively). (B) Principal components analysis (PCA) showing the 183 inbred lines spatially distributed in relation to the first two main components. Values in parentheses indicate the percentage of variation explained by each main component.

According to the method proposed by Evanno et al. [43], linked to the STRUCTURE results, the 183 maize genotypes were divided into two genetically differentiated groups: field corn (98 genotypes) and popcorn (85 genotypes) (Fig 1A). These results were similar to what was visualized with the first two primary components of the PCA method (Fig 1B) and neighbor joining dendrogram (Fig 1A). The axes PC1 and PC2 explained 11.6% and 3.4% of the variation in the genotypic data, respectively, and were the first axes that separated most of the inbred lines.

### Linkage disequilibrium (LD)

**Fig 2:**
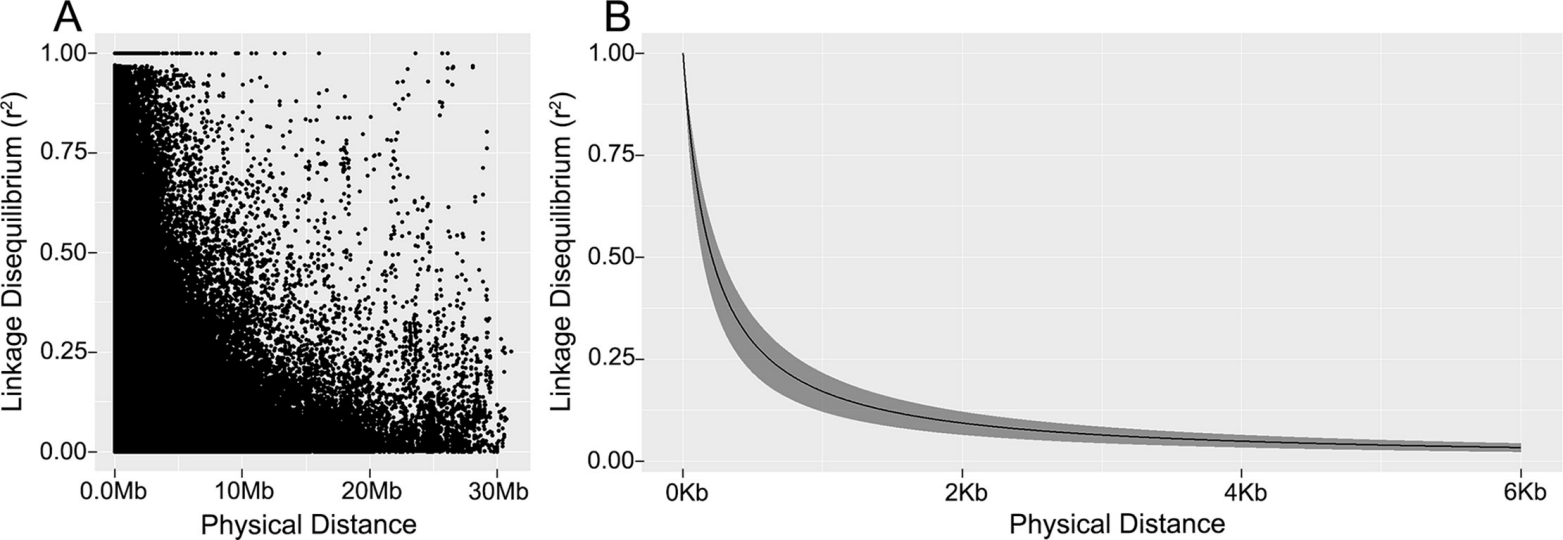
Linkage disequilibrium (LD) in tropical maize inbred lines. **(**A) LD between all pairs of SNP (38K) (r^2^) and their distance (Mb). (B) Pattern of LD decay in the 10 chromosomes, with chromosomes 7 and 5 having the fastest and slowest decay (lower and upper margin, respectively).

The pattern of LD was estimated at the level of the complete genome and for each chromosome, considering a high density of SNP markers (38K). The LD presented a rapid decrease (Fig 2), showing variations among the different chromosomes, with chromosome 7 decreasing the fastest and chromosome 5 decreasing the slowest. The threshold value (*r*^*2*^) for which the LD is likely to be caused by a real physical linkage [36] was 0.19 for all of the chromosomes. LD varied, with values of 0.6 to 0.7 kb on chromosomes 7 and 10, 0.8 to 0.82 kb on chromosomes 1 and 2, 0.84 to 0.92 kb on chromosomes 3 and 9, and 1.01 to 1.2 kb on chromosomes 4, 5, 6 and 8. The LD of the complete genome was 0.90 kb, which coincides with the average of all the chromosomes (0.92 kb).

### Genome-wide association study with individual SNP markers

The linear model used in the GWAS considered the matrix information of Q (genetic structure) and K (kinship), to reduce the false positive rate and correct the spurious associations in the analysis [35]. According to the analysis of mixed models, a total of 163 SNPs were identified to be associated with the four traits of interest, 15 of which were associated in both growing seasons and 11 of which were shared among multiple traits. Twenty-five of the detected SNPs were associated with LA, 37 presented significant associations with EH, 24 were associated with PH, and 67 SNP were associated with the EH/PH ratio. The information regarding the SNPs detected in this study are summarized in Table 2.

**Table 2:** Summary of the associations detected by GWAS, based on individual SNPs, for leaf angle (LA), ear height (EH), plant height (PH) and the EH/PH ratio, evaluated in inbred lines of tropical maize over two growing seasons.

Trait	2014–2015			2015–2016		
	N°_s_	Chromosome (N°_s_)	PV (%)	N°_s_	Chromosome (N°_s_)	PV (%)
Architecture						
LA	20	1(2), 2(3), 4(5), 5 (6), 6(1), 7(1) and 8(2)	10.2–22.2	15	1(2), 2(2), 4(4), 5(5), 6(1) and 8(1)	11.1–21.0
Lodging						
PH	9	1(3), 2(1), 3(1), 4(1), 7(1) and 8(2)	7.9–13.6	15	1(3), 2(1), 3(1), 5(2), 6(1), 7(1), 8(1), 9(3) and 10(2)	7.8–15.3
EH	17	1(1), 3(1), 6(1), 7(5), 8(7) and 10(2)	7.1–12.0	20	1(1), 2(1), 3(1), 4(4), 5(2), 6(2), 7(5), 8(1), 9(1) and 19(2)	8.3–15.2
EH/PH	46	1(3), 2(4), 4(9), 5(4), 6(3), 7(5), 8(5), 9(7) and 10(6)	7.0–11.4	21	1(5), 2(1), 4(2), 5(1), 6(1), 7(2), 8(6), 9(1) and 10(2)	8.2–12.0

PV (%): percentage of the phenotypic variation explained by SNP markers; N°_s_: number of significant SNP-based associations.

**Fig 3:**
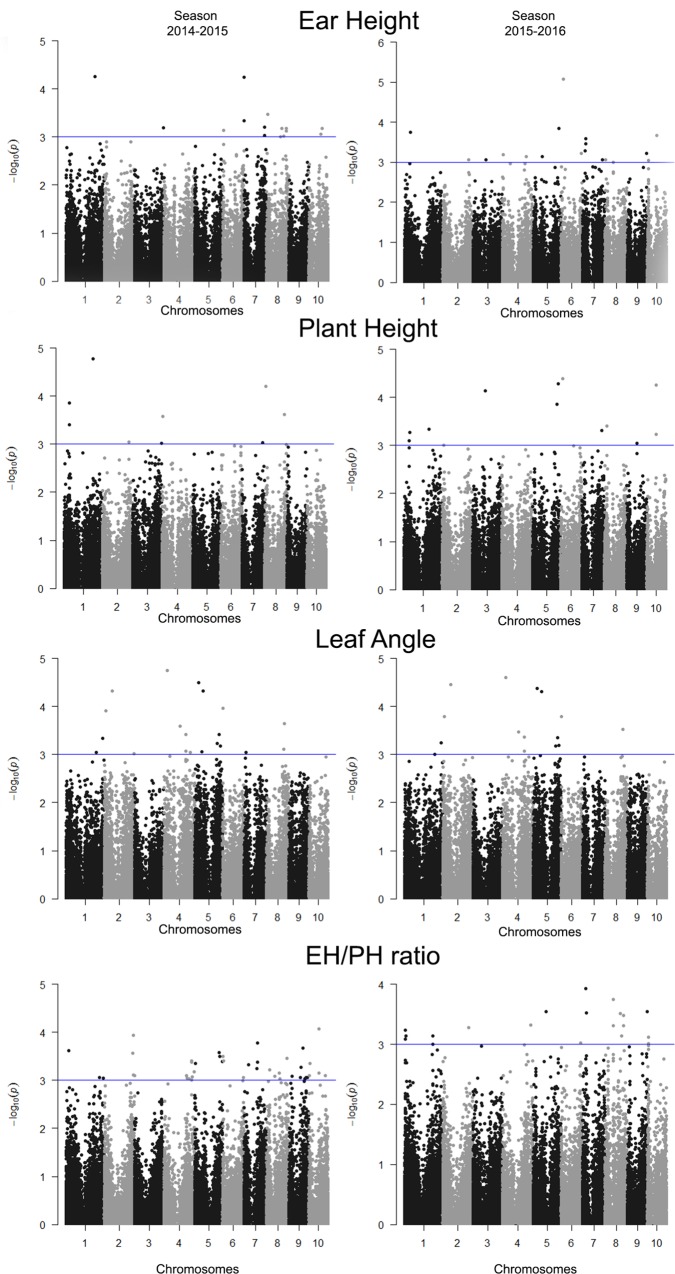
Manhattan plots of the statistical significance of the SNPs associated with the traits leaf angle (LA), ear height (EH), plant height (PH) and EH/PH ratio, evaluated in an association panel of tropical maize during the growing seasons 2014–2015 and 2015–2016. The -log10 (P-values) of the SNPs were plotted for all chromosomes as a whole. The SNPs significantly associated with the traits are above the threshold line (P-value > 1.0 × 10^−3^).

The Fig 3 shows Manhattan plots with the GWAS results. A total of 17 SNPs associated with EH were present in the 2014–2015 growing season, and 20 different SNPs were identified in the following season. Similarly, for LA, 20 and 15 SNPs were associated in the growing seasons 2014–2015 and 2015–2016, respectively. For PH, 9 and 15 SNPs were associated in the growing seasons 2014–2015 and 2015–2016, respectively. For the EH/PH ratio, 46 SNPs were associated in the 2014–2015 growing season, and 21 SNPs were associated in the 2015–2016 season.

The proportion of the phenotypic variation explained by SNPs for LA varied between 10.2 and 22.2%, with SNP S1_317085830, located on chromosome 6, being the trait that presented the greatest effect during the growing season 2014–2015 (Table 2). In general, the *r*^*2*^ values were moderate to relatively high, explaining between 7% (SNP S1_285330124, associated with the EH/PH ratio during the 2014–2015 season, located on chromosome 9) and 22.2% (SNP S1_317085830, associated with LA during the 2014–2015 season) of the phenotypic variation (Table 3). The *r*^*2*^ values in this study were relatively higher than those found in the literature for these traits [28]. Two of the loci with relatively major effects related to plant lodging, the SNPs S1_333289434 (located on chromosome 6) and S1_81113660 (on chromosome 10), were concomitantly associated with EH and PH in the 2015–2016 growing season, which explained 9.5–15.2% and 10.6–15.3% of the phenotypic variations, respectively. Additionally, other marker-trait associations were jointly identified for PH and EH, such as the SNPs S2_47326011 (bin 1.03), S2_230948259 (bin 1.08), S1_1158043959 (bin 3.04), S1_1280061812 (bin 3.09), S1_1037703810 (bin 5.06), S1_821562289 (bin 7.04), S1_494063499 (bin 8.01) and S1_639521797 (bin 8.06). Similarly, SNP S1_16427856 (bin 10.02) was jointly associated with the traits EH and the EH/PH ratio. These results suggest a possible pleiotropic effect for the traits related to lodging [1].

**Table 3:** Details of the associations with relatively major effects (>15%), detected in a GWAS based on individual SNPs for the traits of leaf angle (LA), ear height (EH), plant height (PH) and the EH/PH ratio and measured in inbred lines of tropical maize during two growing seasons (2014–2015 and 2015–2016).

Season	Trait	Marker	Chr	Position (bp)	Bin	p-value	PV (%)
2014–2015	Architecture						
	LA	S1_1348935692	2	62476194	2.04	4.7E-06	16.5%
	LA	S1_901406458	5	65152212	5.03	4.7E-06	16.9%
	LA	S1_1296983734	2	10524236	2.02	1.2E-05	17.3%
	LA	S1_867050714	5	30796468	5.03	3.1E-06	17.7%
	LA	S1_1648381508	4	124004442	4.05	2.6E-05	17.8%
	LA	S1_1550828517	4	26451451	4.04	1.8E-06	18.6%
	LA	S1_317085830	6	2443523	6.00	1.1E-05	22.2%
2015–2016	Architecture						
	LA	S1_901406458	5	65152212	5.03	4.9E-06	16.8%
	LA	S1_1296983734	2	10524236	2.02	1.6E-05	16.8%
	LA	S1_1348935692	2	62476194	2.04	3.5E-06	17.0%
	LA	S1_867050714	5	30796468	5.03	4.3E-06	17.1%
	LA	S1_1648381508	4	124004442	4.05	3.4E-05	17.3%
	LA	S1_1550828517	4	26451451	4.04	2.5E-06	17.9%
	LA	S1_317085830	6	2443523	6.00	1.6E-05	20.9%
	Lodging						
	PH	S1_81113660	10	73141785	10.03	5.5E-05	15.3%
	EH	S1_333289434	6	18647127	6.01	8.3E-06	15.2%

PV (%): percentage of the phenotypic variation explained by SNP markers

In this study, more than four associations per bin were found in the chromosomal bins 1.03, 1.08, 4.07, 4.08, 4.09, 5.03, 5.05, 6.00, 7.02, 7.04, 8.03, 8.04, 8.05, 8.06, 9.03 and 10.03. Bin 1.03 contained a large number of SNPs (9) associated with the traits of interest, six of which are associated with PH, two with EH, and one with LA. Notably, this genomic region had associations for three of the traits, suggesting a possible link among them.

All of the associations detected in the PH, EH and the EH/PH ratio were year-specific (they are present in a particular growing season), while for LA, 75% of the associations were present in both years. This result indicates that most of the QTLs for LA were stable in both growing seasons. This result is in accordance with the AIC and BIC information criteria, which point to the significant presence of a genotype-environment interaction (line x growing season) for PH, EH and the EH/PH ratio. However, the model that did not include the genotype-environment interaction (M2) was a better fit for the LA data, according to the AIC and BIC, indicating that the genotype-environment interaction does not provide information to the complete model [55]. Thus, the inbred lines did not show interaction with the growing seasons, displaying stability in LA, and consequently, in the associations identified through GWAS.

### Haplotypes associated with complex traits

**Fig 4:**
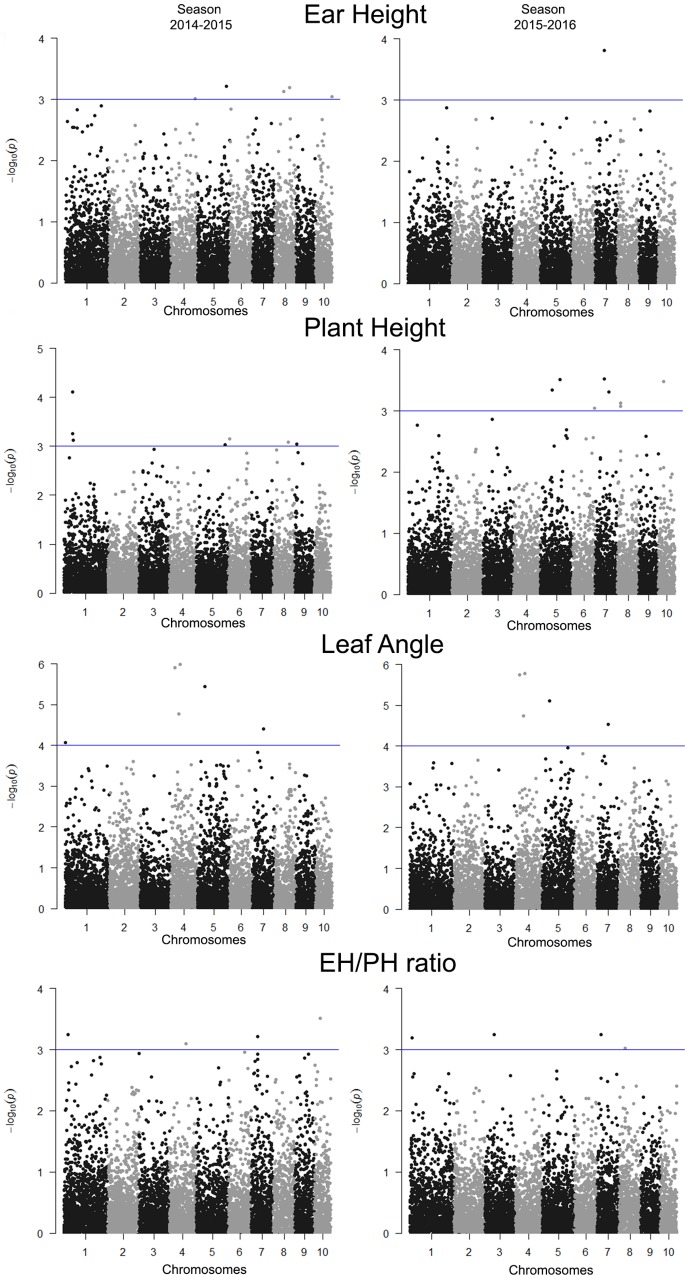
Manhattan plots of the statistical significance of the haplotypes associated with the traits leaf angle (LA), ear height (EH), plant height (PH) and EH/PH ratio, evaluated in an association panel of tropical maize during the growing seasons 2014–2015 and 2015–2016. The -log10 (P-values) of the haplotypes were plotted for all chromosomes as a whole. The haplotypes significantly associated with the traits are above the threshold line (P-value > 1.0 × 10^−3^).

A total of 7,831 haplotypes (each containing between 2 and 20 SNPs) were identified in the 10 maize chromosomes. Of these haplotypes, 55% contained two SNPs, 23% contained three SNPs, 10% contained four SNPs, and 12% contained five to 20 SNPs. In turn, the complete genome association analysis based on haplotypes identified 40 blocks that were significantly associated with the traits of interest, of which 6 were associated with EH, 11 with LA, 15 with PH and 8 with the EH/PH ratio (Table 4) (Fig 4). Three haplotypes (*hap*EH5.07, *hap*EH7.02 and *hap*EH8.06) were concomitantly associated with EH and PH (in the same year of measurement), suggesting a possible pleiotropic effect. In addition, 83% (5/6) of the haplotypes detected for the LA trait were present in both growing season, suggesting the possible stability of these associations.

**Table 4:** Summary of the haplotypes associated with the traits of leaf angle (LA), ear height (EH), plant height (PH) and the EH/PH ratio, measured in inbred lines of tropical maize during two growing seasons (2014–2015 and 2015–2016).

Trait	2014–2015			2015–2016		
	N°_h_	Chromosome (N°_h_)	PV (%)	N°_h_	Chromosome (N°_h_)	PV (%)
Architecture						
LA	6	1(1), 4(3), 5(1) and 7(1)	16–29	5	4(3), 5(1) and 7(1)	18–28
Lodging						
PH	7	1(3), 5(1), 6(1), 8(1) and 9(1)	8–17	8	5(2), 6(1), 7(2), 8(2) and 10(1)	8–17
EH	5	4(1), 5(1), 8(2) and 10(1)	9–18	1	7(1)	15
EH/PH	4	1(1), 4(1), 7(1) and 10(1)	8–16	4	1(1), 3(1), 7(1) and 8(1)	10–16

PV (%): percentage of the phenotypic variation explained by haplotype blocks; N°_h_: number of significant haplotype-based associations.

The haplotypes *hap*EH10.07 and *hap*EH4.09 showed differences greater than 40% among their allelic variants for the trait EH. In effect, individuals that presented the "b" allele of the haplotype *hap*EH10.07 had an ear height 62% and 93% higher than those of individuals with the alleles "a" and "c", respectively. On the other hand, individuals with the "d" allele of haplotype *hap*EH4.09 presented 43%, 88% and 48% higher EH than those of individuals with the alleles "a", "b" and "c", respectively. These results show the ability of these haplotypes to differentiate individuals with higher ear height. For loci with possible pleiotropic effects, individuals that presented the "a" and "c" alleles of the haplotype *hap*EH7.02-*hap*PH7.02 showed significant differences compared with individuals with the "b" allele, for both traits (EH and PH) in the 2015–2016 season. Indeed, plants that presented the "a" and "c" alleles (haplotype *hap*PH7.02) were 19% and 23% taller, respectively, than those of individuals with the "b" allele, and had 29% and 42% higher EH (*hap*EH7.02), respectively, than individuals with the "b" allele. In addition, the blocks *hap*EH5.07-*hap*PH5.07 and *hap*EH8.06-*hap*PH8.06 also presented differences between their allelic variants for both traits (EH and PH) during the 2014–2015 growing season. Plants that presented the allele "b" in the haplotype *hap*PH5.07 were 15% and 22% taller than individuals with the allelic variants "a" and "c", while for EH, plants that presented the "b" allele (*hap*EH5.07) had 22% and 33% higher EH than individuals with the "a" and "c" alleles. Similarly, individuals with the "c" and "d" alleles of the haplotype *hap*PH8.06 were 10% to 14% taller, and had 9% to 21% higher EH than those of individuals with the "a" and "b" alleles.

A total of 38% of the haplotypes significantly associated with a certain trait contained one or more SNPs that were previously detected during the association analysis using individual markers. This result shows that the haplotype analysis allowed the identification of genetic regions that were not detected by GWAS with the use of individual markers.

The phenotypic variation explained by the haplotypes varied between 16% and 29% for LA, between 8% and 17% for PH, between 9% and 18% for EH, and between 8% and 16% for the EH/PH ratio (Table 4). As in the analysis using individual SNPs, the LA trait presented the highest values for *r*^*2*^ (Table 5), in which two haplotypes can explain more than 23% of the phenotypic variation (haplotypes *hap*LA4.2 and *hap*LA1.1, located on chromosome 4; Table 5). These values are slightly higher than those observed in the analysis of individual SNPs. Although the haplotypes encompass a larger genetic region (due to the grouping of SNPs), 45% of these did not increase the phenotypic variation explained in comparison to the SNPs that compose them; for example, the haplotype *hap*PH9.02 explained 8% of the phenotypic variation, while the sum of its SNPs explained 14%. However, 55% of the haplotypes explain a phenotypic variation greater than the sum of the SNPs that compose them; for example, the haplotype *hap*4.05B explains 23% of the variation, while the sum of its 3 SNPs only explains 6%. The latter example shows the potential presented by the use of haplotypes over individual SNPs to increase the phenotypic variation explanations for a trait.

**Table 5:** Haplotype-based associations with relatively major effects (>15%) for the traits of leaf angle (LA), ear height (EH), plant height (PH) and the EH/PH ratio, measured in inbred lines of tropical maize during two growing seasons.

Season	Trait	Marker	Chr	Position (bp)	Bin	p-value	PV (%)
2014–2015	Architecture						
	LA	*hap*LA1.01	1	2993589–2993592	1.01	8.5E-05	16.3%
	LA	*hap*LA7.03	7	132216972–132217768	7.03	4.0E-05	17.0%
	LA	*hap*LA4.05A	4	74484290–74484291	4.05	1.7E-05	18.3%
	LA	*hap*LA5.03	5	30796468–30796505	5.03	3.5E-06	19.9%
	LA	*hap*LA4.05B	4	118922762–118922809	4.05	1.0E-06	23.3%
	LA	*hap*LA4.04	4	26450981–26451472	4.04	1.2E-06	28.8%
	Lodging						
	PH	*hap*PH6.00	6	8145260–8148648	6.00	7.0E-04	15.0%
	PH	*hap*PH8.06	8	155471554–155472264	8.06	8.1E-04	17.4%
	EH	*hap*EH8.06	8	155471554–155472264	8.06	6.5E-04	18.0%
	EH	*hap*EH4.09	4	235925519–235927228	4.09	9.8E-04	18.4%
	EH/PH	*hap*EH/PH10.03	10	79150221–79159436	10.03	3.1E-04	15.9%
2015–2016	Architecture						
	LA	*hap*LA7.03	7	132216972–132217768	7.03	2.9E-05	17.8%
	LA	*hap*LA4.05A	4	74484290–74484291	4.05	1.8E-05	18.1%
	LA	*hap*LA5.03	5	30796468–30796505	5.03	7.9E-06	18.4%
	LA	*hap*LA4.05B	4	118922762–118922809	4.05	1.6E-06	22.8%
	LA	*hap*LA4.04	4	26450981–26451472	4.04	1.8E-06	28.3%
	Lodging						
	PH	*hap*PH7.02	7	127179852–127179870	7.02	3.0E-04	15.2%
	PH	*hap*PH10.03	10	73141503–73141785	10.03	3.4E-04	16.0%
	PH	*hap*PH6.07	6	166163113–166163458	6.07	9.0E-04	17.0%
	EH	*hap*EH7.02	7	127179852–127179870	7.02	1.5E-04	15.1%
	EH/PH	*hap*EH/PH1.01	1	9391163–9391230	1.01	6.4E-04	16.2%

PV (%): percentage of the phenotypic variation explained by haplotype blocks

### Gene annotation based on individual SNPs and haplotypes

Based on the physical position (reference genome B73) of the significantly associated SNPs and haplotypes, according to GWAS, 122 SNPs and 31 haplotypes are close to candidate genes. Twenty of these SNPs, and eight haplotypes were associated with more than one trait or one season at a time. Moreover, some SNPs and haplotypes (20) are close to same candidate genes. Therefore, only 95 unique candidate genes were found in the present analysis. Six of these candidate genes are of particular interest because of their proven participation in the expression of the traits of interest (i.e., *GRMZM2G141386*, *GRMZM2G130675*, *GRMZM2G104262*, *GRMZM5G845755*, *AC205122*.*4_FG003* and *GRMZM2G042429*).

For LA, the *GRMZM2G104262* gene has an ortholog in *Arabidopsis thaliana* that encodes cryptochrome 1 (CRY1), which, at the low wavelengths of blue light, establishes the rapid induction of the hyponastic growth of the petiole [58], independent of ethylene [58, 59]. Moreover, Wu and Yang [60] demonstrated that the overexpression of *CRY1* positively regulates systemic acquired resistance (SAR) and positively regulates the expression of R protein, which mediates resistance to *Pseudomonas syringae*, limiting the growth of bacteria in *Arabidopsis*.

For EH, the *AC205122*.*4_FG003* gene encode for an uncharacterized protein in maize; however, its homolog in *Arabidopsis* encodes the protein C2-domain ABA-related 5 (CAR5), which is related to the sensitivity to abscisic acid (ABA) [61]. In fact, CAR5 mutants showed reduced sensitivity to ABA and rapid growth [61]. In addition, the *GRMZM5G845755* gene encodes the subunit protein kinase alpha 4 (CKA4), a component of protein kinase CK2 (a structural tetramer complex), which has properties very similar to those described in other plants [62]. Lee et al. [63] demonstrated that this protein appears to be involved in plant growth. Further, Wang et al. [64] reported that, in *Arabidopsis*, a knockout mutant of *CKA4* exhibits defects both in the development and elongation of the hypocotyl, delaying growth in general.

For marker-PH associations, the *GRMZM2G141386* gene encodes a putative RNA-binding protein (ARP1). However, Makabe et al. [65] characterized this protein as a disease resistance activator and a plant growth repressor in *Nicotiana tabaccum*. The genes *GRMZM2G042429* (close marker-PH associations) and *GRMZM2G130675* (EH/PH ratio), express small auxin-up RNA 52 (SAUR52) and SAUR1, respectively, two members of the SAUR family, which is composed of genes with early responses to auxin and are key effectors of the hormonal and environmental signals that regulate the growth and development of plants [66]. In soybean and *Arabidopsis thaliana*, these proteins can promote the elongation of the hypocotyl when stimulated by auxins [67, 68].

## Discussion

### Genotype-environment interaction (locus-environment)

For LA, 15 SNPs (75%) and 5 associated haplotypes (83%) were identified during both growing seasons. The selection of the model that does not consider the genotype-environment interaction as the best fit for LA, according to the AIC and BIC criteria, supports the stability resulting from LA associations. However, for the traits PH, EH and the EH/PH ratio, the model with the best fit, according to AIC and BIC, includes the effects of the genotype-environment interactions (the complete model), indicating that the response of the inbred lines was dependent on the growing season. This result allows us to explain the absence of stable SNPs or haplotypes during both seasons. Our results indicate that the genotype-environment interactions were important for the study of traits related to plant lodging. In addition, this result could be influenced by differences between the two growing seasons, as both PH and EH showed higher average values in the first season, indicating better growth conditions. Asaro et al. [19] mentioned that local soil differences (from the same test) can favor the differential identification of QTLs between two growing seasons, suggesting that the identified associations may be responding to components such as precipitation, temperature or other climatic factors. The lack of stability between the QTLs detected in PH, EH and the EH/PH ratio can be attributed to the environmental variations that occur during the seasons, demonstrating the importance of performing tests over time and/or in different environments [20]. According to Edmeades [18], in many maize populations, there are random events that can cause genotype-year interactions that are difficult to interpret. For this reason, to obtain associations between genetic stability and the high phenotypic variations explained, the same genetic material should be evaluated during different seasons or in different environments.

The growing seasons in which the inbred maize lines were evaluated did not affect the stability of the associations found for LA, which is in accordance with related studies. Maize can be grown in a wide range of conditions, but abiotic stress variables, such as temperature, soil fertility and climatic factors, affect its production [69]. Therefore, the detection of stable SNPs and haplotypes during different growing seasons can be very useful for breeding programs that seek to identify the best varieties that can adapt to changes in environmental conditions.

### Haplotypes and genetic regions associated with complex traits

The rapid decay in LD is typical in the tropical maize germplasm [21, 70]. Remington et al. [71] and Yan et al. [72] reported that LD decays within the range of 0.1 to 10 kb, depending on the population and the genetic region studied, and always decays faster in tropical maize [70]. The size of the haplotypes is closely related to the degree of LD present in the population studied [73], in which a rapid decay of LD produces smaller haplotypes because a smaller number of loci will be linked [73, 74]. The majority of the haplotype blocks were formed with two or three SNPs (78%), due to the rapid decay of the LD (0.9 kb). Lorenz et al. [78] and Contreras-Soto et al. [22] demonstrated that the use of haplotype information can be beneficial when identifying marker-phenotype associations and can offer advantages for the genetic dissection of loci underlying complex traits. The use of haplotype-based analysis reduces the number of multiple comparisons (or multiple testing), compared to individual SNP-based association analysis, as haplotypes can group SNPs from the LD pattern observed in the data. The dimension reduction, from a biological perspective (haplotypes) becomes more relevant when considering the new genomic data platforms (high-density genomic data), thereby increasing the possibility of finding genomic regions controlling the variation of a trait.

A total of 38% of the significantly associated haplotypes contained one or more SNPs that were previously detected in the GWAS, indicating the advantage that haplotypes have in the detection of multiple DNA variants [22]. The selected SNP-GWAS and haplotype-GWAS thresholds were based on the Bonferroni correction method, these thresholds were equivalent to p-values = 2.6x10^-6^ and 1.3x10^-5^, respectively. Although the loci identified did not surpass Bonferroni thresholds, the p-values in both analyses were significant (p-values < 1×10^−3^), in which 73% of the loci had p-values lower than 8.5x10^-5^ (and 41% lower than 8.3x10^-6^). It is worth noting that 16% of DNA variants were consistently detected in both methods, confirming the high reliability of the detected loci. In this study, the associated haplotypes explain a greater proportion of the phenotypic variability of a trait than the SNPs that compose it. For example, the haplotypes *hap*LA4.05B and *hap*EH4.09 explained 23% and 18% of the phenotypic variation for LA and EH, respectively, while the sum of its 3 SNPs only accounted for 6% and 4%, respectively. Moreover, the haplotype *hap*EH4.09 showed significant differences (up to 88%) among its allelic variants, with individuals containing the “d” allele being superior to those with the other alleles. This result indicates the potential utility of haplotypes to explain the phenotypic variation associated with a trait, an aspect highlighted by Contreras-Soto et al. [22], Chen et al. [30] and Barendse [79].

The EH and PH traits had a high and positive correlation (r = 0.89), which is in accordance with previous studies [1, 12]. However, the EH/PH ratio had a greater correlation with EH (r = 0.80) than with PH (r = 0.42), indicating that the variation in the EH/PH ratio is dominated primarily by changes in EH. Therefore, it is possible to observe that, in the same genomic regions, markers can be found that are significantly associated with both PH and EH, and with EH/PH ratio and EH [1, 80]. In the present study, three haplotypes and ten SNPs showed significant associations with PH and EH concomitantly, whereas only one SNP was simultaneously associated with the EH/PH ratio and EH. This suggests a common genetic control for these traits, which could result in possible pleiotropic effects for these loci [1].

The pleiotropy among loci associated with the traits of plant lodging was corroborated by a multivariate Bayesian regression [53]. As PPA combines the evidence in the observed association data (BF) with the prior probability (π) that an SNP is associated with a given trait, we regarded that loci with values of APP > 0.9 are truly associated with a given phenotype. This value of PPA corresponds to a log10 (BF) > 3.95, which is higher than previous association studies [75, 76, 77].

The PPA values of 0.924 and 0.994, for SNPs S2_230948259 and S1_81113660, respectively (Table 6) provide convincing evidence of the association of these two loci with more than one phenotype (EH and PH). Similarly, the high values of log10 (BF) (> 3.95) can be considered as strong evidence against the null hypothesis of no association. This Bayesian analysis indicates that only two loci presented a significant pleiotropic effect.

**Table 6:** Analysis of pleiotropic loci with their respective the p-values for traits related to plant lodging. The Bayes Factor (BF), posterior odds (PO) and the corresponding posterior probability of association (PPA) for two values of the prior probability are shown.

Loci		Season	p-values			Log10(BF)	PO	PPA
			EH	PH	EH/PH			
SNP	S1_821562289	1	6.2×10^−4^	9.3×10^−4^	-	0.4	0.000	0.000
	S1_494063499	1	3.4×10^−4^	6.2×10^−5^	-	0.9	0.008	0.008
	S1_639521797	1	7.5×10^−4^	2.4×10^−4^	-	1.6	0.037	0.035
	S1_1280061812	1	6.4×10^−4^	9.5×10^−4^	-	2.0	0.107	0.097
	S2_230948259	1	5.5×10^−5^	1.7×10^−5^	-	4.1	12.15	0.924
	S2_47326011	2	1.8×10^−4^	5.4×10^−4^	-	0.8	0.007	0.007
	S1_333289434	2	8.3×10^−6^	4.1×10^−5^	-	1.8	0.069	0.064
	S1_1158043959	2	8.6×10^−4^	7.4×10^−5^	-	3.0	1.033	0.508
	S1_1037703810	2	1.4×10^−4^	5.2×10^−5^	-	3.1	1.186	0.543
	S1_81113660	2	2.2×10^−4^	5.5×10^−5^	-	5.2	153.93	0.994
	S1_16427856	2	9.0×10^−4^	-	7.6×10^−4^	-0.3	0.000	0.000
Haplotype	*hap*EH5.07-*hap*PH5.07	1	6.1×10^−4^	9.2×10^−4^	-	0.7	0.005	0.005
	*hap*EH8.06-*hap*PH8.06	1	6.5×10^−4^	8.1×10^−4^	-	2.2	0.169	0.144
	*hap*EH7.02-*hap*PH7.02	2	1.5×10^−4^	3.0×10^−4^	-	-0.4	0.000	0.000

P-values are shown only in the traits associated with the indicated loci

In the present study, the SNPs and haplotypes associated with PH, EH and the EH/PH ratio were distributed in all of the chromosomes, confirming their quantitative architecture, while those associated with LA were only found in chromosomes 1, 2, 4, 5, 6, 7 and 8. Several previous studies in maize that used biparental populations and recombinant inbred lines for associative mapping have reported similar genomic localizations for marker-trait associations. Weng et al. [7], for example, found 27 QTLs in bin 1.03, 6 associated with PH and 21 with EH. In addition, Li et al. [1] reported three QTLs in bin 2.07 associated with EH, and Pan et al. [81] identified four QTLs, three associated with PH (bins 3.09, 8.06 and 9.03) and one with EH (bin 6.06).

In the present study, several hotspots (containing more than three SNPs or haplotypes) were identified in the chromosomal bins 1.03, 9.03 and 10.03 for PH, bins 4.08, 7.00, 7.02, 8.04 and 8.06 for EH, bins 4.05 and 5.03 for the LA trait, and bins 1.01, 4.09, 7.02 and 8.03 for the EH/PH ratio. In addition, the SNPs S2_47326011 and S1_639521797 that were concomitantly associated with PH and EH were located in bins 1.03 and 8.06, respectively, which are two hotspots related to the lodging of plants. At the haplotype level, *hap*EH8.06-*hap*PH8.06, which is also concomitantly associated with both EH and PH traits, is located in bin 8.06. Coincidentally, Weng et al. [7] reported QTLs shared between EH and PH in bin 1.03, suggesting that these regions may contain genetic mechanisms that control both traits.

To date, several QTL mapping studies have been reported for plant lodging and architecture [16, 82, 83, 84, 85]. However, only a few have reported QTLs or associations with relatively major effects. In this study, a total of 9 SNPs and 14 haplotypes linked to QTLs were able to explain a significant percentage of the phenotypic variation (>15%) in the lodging and architecture traits of the plant (Table 3 and Table 5). These associations were found in chromosomes 1, 2, 4, 5, 6, 7, 8 and 10, which agrees with previously reported studies. For traits related to the lodging of plants, several studies have identified loci with major effects; for example, Zhang et al. [84] identified a QTL explaining much of the phenotypic variation for PH on chromosome 4, while Zhu et al. [85] found major effect QTLs for EH and PH that were located on chromosomes 1, 8, 9 and 10. In this study, one SNP (S1_81113660) and three haplotypes (*hap*EH8.06, *hap*PH8.06 and *hap*PH10.03) of greater effect coincide with the QTLs described by Zhu et al. [85] on chromosomes 8 and 10. For LA, QTLs have been identified that contribute in large part to the phenotypic variation; for example, Ku et al. [16] identified two QTLs that explain more than 17% of the variation, Wang et al. [82] reported only one major effect marker on chromosome 7, and Zhang et al. [83] found a QTL explaining 36.82% of the phenotypic variation. Among the major effect QTLs identified in this study, the haplotype *hap*LA1.01 (16.3%) and SNP S1_1296983734 (16.8%) are adjacent to the major effect QTLs found by Ku et al. [16] on chromosomes 1 and 2, respectively, while the haplotype *hap*LA7.03 (17%) is adjacent to qLAa7-1, described by Wang et al. [82]. Likewise, two SNPs, S1_1648381508 (17.8%) and S1_1550828517 (18.6%), and three haplotypes, *hap*LA4.05A (18.1%), *hap*LA4.05B (22.8%) and *hap*LA4.04 (28.3%), were located adjacent to QTL qLA4-1 (36.82%), detected by Zhang et al. [83], a key locus that explains much of the phenotypic variation for LA. Importantly, the major effect QTLs identified for LA *(hap*LA4.05B = 23.3% and *hap*LA4.04 = 28.8%) were located on chromosome 4, which is consistent with the study by Zhang et al. [83]. This result confirms that chromosome 4 plays a key role in the variation of LA and should be considered in breeding programs of maize.

Our results indicate that the traits of interest (plant lodging and architecture) are controlled by multiple genes, with variable contributions to phenotypic expression. In this work, the first major effect haplotypes associated with leaf angle are presented, representing a fundamental aspect for architecture of maize and marker-assisted selection, and which appear to be stable between growing seasons. In general, the haplotypes that are significantly associated with traits related to plant lodging and architecture explain a greater proportion of the phenotypic variance in comparison to the associated SNPs, which supports the hypothesis that using haplotypes in association studies allows the identification of genomic regions responsible for controlling a large part of the variation in the traits of interest.

### Annotated genome data

Maize is an ideal biological system for the application of GWAS through panels of inbred lines [21] because there is a high-quality reference genome that allows the generation of enough informative markers [21, 33]. In addition, one of the advantages of GWAS is that it allows high-resolution mapping. For the identification of genomic annotations for the traits of interest, we considered a 0.9 kb window (determined by the LD pattern) upstream and downstream of the significant associations. Based on this principle, annotations of 126 unique candidate genes were found, of which only six genes were considered to be biologically important due to their proven participation in the studied traits (i.e., the genes *GRMZM2G141386*, *GRMZM2G130675*, *GRMZM2G104262*, *GRMZM5G845755*, *AC205122*.*4_FG003* and *GRMZM2G042429*) [58, 60, 61, 62, 63, 64, 65, 66, 67, 68].

LA presented a gene orthologous to *GRMZM2G104262* in *Arabidopsis*, which encodes CRY1. According to Millenaar et al. [58], CRY1 has been reported to have two positively correlated functions with light intensity: the rapid induction of the hyponastic growth of the petiole at low levels of low blue light wavelength, and the positive regulation of *Pseudomonas syringae* resistance [60]. In turn, Joshi and Chand [86] showed a positive correlation between LA and spot blotch resistance, caused by *Bipolaris sorokiniana* in wheat, in which individuals with straight or semi-straight leaves presented a lower incidence of the disease compared to individuals with droopy leaves. These results would justify the double function observed in CRY1 and suggest that LA can influence the incidence of a disease. It is therefore necessary to perform investigations that elucidate the genetic structure of LA in maize and its degree of association with diseases.

The genes *AC205122*.*4_FG003* and *GRMZM5G845755* are found in genomic regions associated with EH and encode polypeptides of unknown function. However, these genes have orthologs in *Arabidopsis* that express the CAR5 and CKA4 proteins, which have been associated with hypocotyl elongation and plant growth [61, 63, 64]. Similarly, for PH and the EH/PH ratio, orthologs for the *GRMZM2G042429* and *GRMZM2G130675* genes, respectively, encode SAUR52 and SAUR1 proteins that can promote hypocotyl elongation through auxin stimulation [67, 68]. Auxin is a key plant growth hormone, which regulates processes such as cell elongation, division, differentiation and morphogenesis during the growth and development of the plant [87]. SAUR proteins participate in the auxin early response, and play key roles in hormonal and environmental signals that regulate the growth and development of plants [66].

In this study, some haplotypes contained SNPs that were not detected in the GWAS using individual SNP markers. This result represents an advantage of using haplotypes over individual SNPs in the detection of multiple DNA variants. Another advantage of haplotypes is that they present multiple allelic variants, which could facilitate the search for loci that affect the gene expression of a certain trait. Finally, the use of the haplotypes and SNPs identified in this study could increase the efficiency of maize breeding programs, based on important determinants of plant lodging and architecture.

## Supporting information

S1 Table Detailed information of SNPs used in the study (SNP name, chromosome, physical position (pb) and polymorphic alleles, recorded in the maize genome AGPV3 (B73 RefGen_v3), available in the MaizeGDB database (http://www.maizegdb.org//). (XLSX)

S2 Table Detailed information of haplotypes blocks determined in this study. Data include the chromosome, the start and end positions of the haplotype block in pb, the size of the haplotype block, and the number of SNPs in the haplotype block. Based in the MaizeGDB database (http://www.maizegdb.org//).
(XLSX)

S3 Table Summary of statistical analysis for key traits of plant lodging and architecture measured in a two growing seasons (2014–2015 and 2015–2016). (XLSX)

## References

[1] Li X, Zhou Z, Ding J, Wu Y, Zhou B, Wang R, et al Combined linkage and association mapping reveals QTL and candidate genes for plant and ear height in maize. Front. Plant Sci. 2016; 7: 833 10.3389/fpls.2016.00833 27379126 PMC4908132

[2] De la Barrera E, Martínez RO. Socio-ecological considerations on the persistence of Mexican heirloom maize. Maydica. 2018; 61(4): 10.

[3] Yang X, Gao S, Xu S, Zhang Z, Prasanna BM, Li L, et al Characterization of a global germplasm collection and its potential utilization for analysis of complex quantitative traits in maize. Mol. Breeding. 2011; 28(4): 511–526.

[4] Băşa AG, Ion V, Dumbravă M, Temocico G, Epure LI, Ştefan D. Grain yield and yield components at maize under different preceding crops and nitrogen fertilization conditions. Agric. Agric. Sci. Proc. 2016; 10: 104–111.

[5] Fischer RA, Edmeades GO. Breeding and cereal yield progress. Crop Sci. 2010; 50: S–85.

[6] Buescher EM, Moon J, Runkel A, Hake S, Dilkes BP. Natural Variation at *sympathy for the ligule* Controls Penetrance of the Semidominant *Liguleless narrow-R* Mutation in *Zea mays*. G3-Genes Genom. Genet. 2014; 4(12): 2297–2306. 10.1534/g3.114.014183 PMC4267926 25344411

[7] Weng J, Xie C, Hao Z, Wang J, Liu C, Li M, et al Genome-wide association study identifies candidate genes that affect plant height in Chinese elite maize (*Zea mays* L.) inbred lines. PLoS One. 2011; 6(12): e29229 10.1371/journal.pone.0029229 22216221 PMC3247246

[8] Kong F, Zhang T, Liu J, Heng S, Shi Q, Zhang H, et al Regulation of Leaf Angle by Auricle Development in Maize. Mol. Plant. 2017; 10(3): 516–519. 10.1016/j.molp.2017.02.001 28216423

[9] Ding J, Zhang L, Chen J, Li X, Li Y, Cheng H, et al Genomic dissection of leaf angle in maize (*Zea mays* L.) using a four-way cross mapping population. PloS one. 2015; 10(10): e0141619 10.1371/journal.pone.0141619 26509792 PMC4625009

[10] Peiffer JA, Romay MC, Gore MA, Flint-Garcia SA, Zhang Z, Millard MJ, et al The genetic architecture of maize height. Genetics. 2014; 196(4): 1337–1356. 10.1534/genetics.113.159152 24514905 PMC3982682

[11] Abdel‐Ghani AH, Hu S, Chen Y, Brenner EA, Kumar B, Blanco M, et al Genetic architecture of plant height in maize phenotype‐selected introgression families. Plant Breeding. 2016; 135(4): 429–438.

[12] Cai H, Chu Q, Gu R, Yuan L, Liu J, Zhang X, et al Identification of QTLs for plant height, ear height and grain yield in maize (*Zea mays* L.) in response to nitrogen and phosphorus supply. Plant breeding. 2012; 131(4): 502–510.

[13] Souza ARR, Vieira Miranda G, Gonzaga Pereira M, Vagno de Souza L, Lopes Ferreira P. Agronomic performance of white maize landrace in different environmental conditions. Revista Ceres. 2008; 55(6).

[14] Yang N, Lu Y, Yang X, Huang J, Zhou Y, Ali F, et al Genome wide association studies using a new nonparametric model reveal the genetic architecture of 17 agronomic traits in an enlarged maize association panel. PLoS Genet. 2014; 10(9): e1004573 10.1371/journal.pgen.1004573 25211220 PMC4161304

[15] Tian F, Bradbury PJ, Brown PJ, Hung H, Sun Q, Flint-Garcia S, et al Genome-wide association study of leaf architecture in the maize nested association mapping population. Nat. Genet. 2011; 43(2): 159 10.1038/ng.746 21217756

[16] Ku LX, Zhao WM, Zhang J, Wu LC, Wang CL, Wang PA, et al Quantitative trait loci mapping of leaf angle and leaf orientation value in maize (*Zea mays* L.). Theor. Appl. Genet. 2010; 121(5): 951–959. 10.1007/s00122-010-1364-z 20526576

[17] Miles C, Wayne M. Quantitative trait locus (QTL) analysis. Nat. Educ. 2008; 1(1): 1–6.

[18] Edmeades GO. Progress in achieving and delivering drought tolerance in maize: An update. ISAAA: Ithaca, NY 2013; 130.

[19] Asaro A, Ziegler G, Ziyomo C, Hoekenga OA, Dilkes BP, Baxter I. The interaction of genotype and environment determines variation in the maize kernel ionome. G3-Genes Genom. Genet. 2016; 6(12): 4175–4183. 10.1534/g3.116.034827 PMC5144985 27770027

[20] García PJ, Cabrera SR, Pérez AA, Silva RJ, Álvarez RM, Marín CA, et al Estabilidad del rendimiento y potencial agronómico de cultivares de maíz de endospermo normal y QPM en zonas agroecológicas de Venezuela. Agronomía Trop. 2009; 59(4): 433–443.

[21] Coan M, Senhorinho HJ, Pinto RJ, Scapim CA, Tessmann DJ, Williams WP, et al Genome-Wide Association Study of Resistance to Ear Rot by *Fusarium verticillioides* in a Tropical Field Maize and Popcorn Core Collection. Crop Sci. 2018; 58(2): 564–578.

[22] Contreras-Soto RI, Mora F, de Oliveira MAR, Higashi W, Scapim CA, Schuster I. A genome-wide association study for agronomic traits in soybean using SNP markers and SNP-based haplotype analysis. PloS one. 2017; 12(2): e0171105 10.1371/journal.pone.0171105 28152092 PMC5289539

[23] Elshire RJ, Glaubitz JC, Sun Q, Poland JA, Kawamoto K, Buckler ES, et al A robust, simple genotyping-by-sequencing (GBS) approach for high diversity species. PloS one. 2011; 6(5): e19379 10.1371/journal.pone.0019379 21573248 PMC3087801

[24] Yu J, Buckler ES. Genetic association mapping and genome organization of maize. Curr. Opin. Biotech. 2006; 17(2): 155–160. 10.1016/j.copbio.2006.02.003 16504497

[25] Yu H, Xie W, Li J, Zhou F, Zhang Q. A whole‐genome SNP array (RICE6K) for genomic breeding in rice. Plant Biotechnol. J. 2014; 12(1): 28–37. 10.1111/pbi.12113 24034357

[26] Mora F, Castillo D, Lado B, Matus I, Poland J, Belzile F, et al Genome-wide association mapping of agronomic traits and carbon isotope discrimination in a worldwide germplasm collection of spring wheat using SNP markers. Mol. Breeding. 2015; 35(2): 69.

[27] Clark AG. The role of haplotypes in candidate gene studies. Genet. Epidemiol. 2004; 27(4): 321–333. 10.1002/gepi.20025 15368617

[28] Xiao Y, Liu H, Wu L, Warburton M, Yan J. Genome-wide association studies in maize: praise and stargaze. Mol. Plant. 2017; 10(3): 359–374. 10.1016/j.molp.2016.12.008 28039028

[29] Abdel-Shafy H, Bortfeldt RH, Tetens J, Brockmann GA. Single nucleotide polymorphism and haplotype effects associated with somatic cell score in German Holstein cattle. Genet. Sel. Evol. 2014; 46(1): 35. 24898131 10.1186/1297-9686-46-35 PMC4078941

[30] Chen J, Shrestha R, Ding J, Zheng H, Mu C, Wu J, et al Genome-wide association study and QTL mapping reveal genomic loci associated with *Fusarium* ear rot resistance in tropical maize germplasm. G3-Genes Genom. Genet. 2016; 6(12): 3803–3815. 10.1534/g3.116.034561 PMC5144952 27742723

[31] Cruz JC, Monteiro JA, Santana DP, Garcia JC, de C. Bahia FGFT, Sans LMA, et al Embrapa milho e sorgo. Sistemas de produção, Embrapa, Sete Lagoas, 2015; 1(9).

[32] Bradbury PJ, Zhang Z, Kroon DE, Casstevens TM, Ramdoss Y, Buckler ES. TASSEL: software for association mapping of complex traits in diverse samples. Bioinformatics. 2007; 23(19): 2633–2635. 10.1093/bioinformatics/btm308 17586829

[33] Schnable PS, Ware D, Fulton RS, Stein JC, Wei F, Pasternak S, et al The B73 maize genome: complexity, diversity, and dynamics. Science. 2009; 326(5956): 1112–1115. 10.1126/science.1178534 19965430

[34] Chen N, Van Hout CV, Gottipati S, Clark AG. Using Mendelian inheritance to improve high-throughput SNP discovery. Genetics, 2014; 198(3): 847–857. 10.1534/genetics.114.169052 25194160 PMC4224174

[35] Yu LX, Zheng P, Bhamidimarri S, Liu XP, Main D. The Impact of Genotyping-by-Sequencing Pipelines on SNP Discovery and Identification of Markers Associated with Verticillium Wilt Resistance in Autotetraploid Alfalfa (*Medicago sativa* L.). Frontiers Plant Sci. 2017; 8. 10.3389/fpls.2017.00089 PMC5293825 28223988

[36] Breseghello F, Sorrells ME. Association Mapping of Kernel Size and Milling Quality in Wheat (*Triticum aestivum* L.) Cultivars. Genetics, 2006; 172(2): 1165–1177. 10.1534/genetics.105.044586 16079235 PMC1456215

[37] Laidò G, Marone D, Russo MA, Colecchia SA, Mastrangelo AM, De Vita P, et al Linkage Disequilibrium and Genome-Wide Association Mapping in Tetraploid Wheat (*Triticum turgidum* L.). PLoS ONE, 2014; 9(4): e95211 10.1371/journal.pone.0095211 24759998 PMC3997356

[38] Marroni F, Pinosio S, Zaina G, Fogolari F, Felice N, Cattonaro F, et al Nucleotide diversity and linkage disequilibrium in *Populus nigra* cinnamyl alcohol dehydrogenase (*CAD4*) gene. Tree Genet. Genomes, 2011; 7(5): 1011–1023.

[39] Barrett JC, Fry B, Maller JDMJ, Daly MJ. Haploview: analysis and visualization of LD and haplotype maps. Bioinformatics. 2004; 21(2): 263–265. 10.1093/bioinformatics/bth457 15297300

[40] Gabriel SB, Schaffner SF, Nguyen H, Moore JM, Roy J, Blumenstiel B, et al The structure of haplotype blocks in the human genome. Science. 2002; 296(5576): 2225–2229. 10.1126/science.1069424 12029063

[41] Endelman JB, Jannink JL. Shrinkage estimation of the realized relationship matrix. G3-Genes Genom. Genet. 2012; 2(11): 1405–1413. 10.1534/g3.112.004259 PMC3484671 23173092

[42] Pritchard JK, Stephens M, Donnelly P. Inference of population structure using multilocus genotype data. Genetics. 2000; 155(2): 945–959. 10835412 10.1093/genetics/155.2.945 PMC1461096

[43] Evanno G, Regnaut S, Goudet J. Detecting the number of clusters of individuals using the software STRUCTURE: a simulation study. Mol. Ecol. 2005; 14(8): 2611–2620. 10.1111/j.1365-294X.2005.02553.x 15969739

[44] Perrier X, Jacquemoud-Collet JP. DARwin software. 2006 Available at (http://darwin.cirad.fr/).

[45] Ferrão LFV, Ferrão RG, Ferrão MAG, Francisco A, Garcia AAF. A mixed model to multiple harvest-location trials applied to genomic prediction in *Coffea canephora*. Tree Genet. Genomes. 2017; 13(5): 95.

[46] Gilmour AR, Gogel BJ, Cullis BR, Welham S, Thompson R. *ASReml user guide release 4*.*1 structural specification*. Hemel Hempstead: VSN International ltd 2015.

[47] Breslow NE, Clayton DG. Approximate inference in generalized linear mixed models. J. Am. Stat. Assoc. 1993; 88(421): 9–25.

[48] Wolfinger R, O'connell M. Generalized linear mixed models a pseudo-likelihood approach. J. Stat. Comput. Sim. 1993; 48(3–4): 233–243.

[49] Arriagada O, Amaral-Júnior AT, Mora F. Thirteen years under arid conditions: exploring marker-trait associations in *Eucalyptus cladocalyx* for complex traits related to flowering, stem form and growth. Breeding Sci. 2018; 68(3): 367–374. 10.1270/jsbbs.17131 30100804 PMC6081299

[50] Myles S, Peiffer J, Brown PJ, Ersoz ES, Zhang Z, Costich DE, et al Association mapping: critical considerations shift from genotyping to experimental design. Plant Cell. 2009; 21(8): 2194–2202. 10.1105/tpc.109.068437 19654263 PMC2751942

[51] R Core Team. R: A language and environment for statistical computing. R Foundation for Statistical Computing, Vienna, Austria 2013; URL http://www.R-project.org/.

[52] Stephens M, Balding DJ. Bayesian statistical methods for genetic association studies. Nat. Rev. Genet. 2009; 10(10): 681 10.1038/nrg2615 19763151

[53] Marchini J, Band G. SNPTEST. 2016; https://mathgen.stats.ox.ac.uk/genetics_software/snptest/snptest.html.

[54] Harper LC, Schaeffer ML, Thistle J, Gardiner JM, Andorf CM, Campbell DA, et al The MaizeGDB Genome Browser tutorial: one example of database outreach to biologists via video. Database, 2011. 10.1093/database/bar016 PMC3096322 21565781

[55] Burnham KP, Anderson DR. Multimodel inference: *understanding AIC and BIC in model selection*. Sociol. Method. Res. 2004; 33(2): 261–304.

[56] Zhang Y, Li YX, Wang Y, Liu ZZ, Liu C, Peng B, et al Stability of QTL across environments and QTL-by-environment interactions for plant and ear height in maize. Agr. Sci. China. 2010; 9(10): 1400–1412.

[57] Brachi B, Morris GP, Borevitz JO. Genome-wide association studies in plants: the missing heritability is in the field. Genome Biol. 2011; 12(10): 232 10.1186/gb-2011-12-10-232 22035733 PMC3333769

[58] Millenaar FF, Van Zanten M, Cox MC, Pierik R, Voesenek LA, Peeters AJ. Differential petiole growth in *Arabidopsis thaliana*: photocontrol and hormonal regulation. New Phytol. 2009; 184(1): 141–152. 10.1111/j.1469-8137.2009.02921.x 19558423

[59] Kozuka T, Horiguchi G, Kim GT, Ohgishi M, Sakai T, Tsukaya H. The different growth responses of the *Arabidopsis thaliana* leaf blade and the petiole during shade avoidance are regulated by photoreceptors and sugar. Plant Cell Physiol. 2005; 46(1): 213–223. 10.1093/pcp/pci016 15659441

[60] Wu L, Yang HQ. CRYPTOCHROME 1 is implicated in promoting R protein-mediated plant resistance to *Pseudomonas syringae* in *Arabidopsis*. Mol. Plant. 2010; 3(3): 539–548. 10.1093/mp/ssp107 20053798

[61] Rodriguez L, Gonzalez-Guzman M, Diaz M, Rodrigues A, Izquierdo-Garcia AC, Peirats-Llobet M, et al C2-domain abscisic acid-related proteins mediate the interaction of PYR/PYL/RCAR abscisic acid receptors with the plasma membrane and regulate abscisic acid sensitivity in *Arabidopsis*. Plant Cell. 2014; 26(12): 4802–4820. 10.1105/tpc.114.129973 25465408 PMC4311195

[62] Riera M, Peracchia G, De Nadal E, Ariño J, Pagès M. Maize protein kinase CK2: regulation and functionality of three β regulatory subunits. Plant J. 2001; 25(4): 365–374. 11260493 10.1046/j.1365-313x.2001.00973.x

[63] Lee Y, Lloyd AM, Roux SJ. Antisense expression of the CK2 α-subunit gene in Arabidopsis. Effects on light-regulated gene expression and plant growth. Plant Physiol. 1999; 119(3): 989–1000. 10069836 10.1104/pp.119.3.989 PMC32112

[64] Wang WS, Zhu J, Zhang KX, Lü YT, Xu HH. A mutation of casein kinase 2 α4 subunit affects multiple developmental processes in *Arabidopsis*. Plant Cell Rep. 2016; 35(5): 1071–1080. 10.1007/s00299-016-1939-5 26883224

[65] Makabe S, Yamori W, Kong K, Niimi H, Nakamura I. Expression of rice *45S rRNA* promotes cell proliferation, leading to enhancement of growth in transgenic tobacco. Plant Biotechnol. 2017; 34(1): 29–38. 10.5511/plantbiotechnology.17.0216a PMC6543702 31275005

[66] Ren H, Gray WM. SAUR proteins as effectors of hormonal and environmental signals in plant growth. Mol. Plant. 2015; 8(8): 1153–1164. 10.1016/j.molp.2015.05.003 25983207 PMC5124491

[67] Hagen G, Guilfoyle T. Auxin-responsive gene expression: genes, promoters and regulatory factors. Plant Mol. Biol. 2002; 49(3–4): 373–385. 12036261

[68] Chae K, Isaacs CG, Reeves PH, Maloney GS, Muday GK, Nagpal P, et al *Arabidopsis SMALL AUXIN UP RNA63* promotes hypocotyl and stamen filament elongation. Plant J. 2012; 71(4): 684–697. 10.1111/j.1365-313X.2012.05024.x 22507274

[69] Ramirez-Cabral NY, Kumar L, Shabani F. Global alterations in areas of suitability for maize production from climate change and using a mechanistic species distribution model (CLIMEX). Sci. Rep-UK. 2017; 7(1): 5910. 10.1038/s41598-017-05804-0 PMC5517596 28724952

[70] Romay MC, Millard MJ, Glaubitz JC, Peiffer JA, Swarts KL, Casstevens TM, et al Comprehensive genotyping of the USA national maize inbred seed bank. Genome Biol. 2013; 14(6): R55 10.1186/gb-2013-14-6-r55 23759205 PMC3707059

[71] Remington DL, Thornsberry JM, Matsuoka Y, Wilson LM, Whitt SR, Doebley J, et al Structure of linkage disequilibrium and phenotypic associations in the maize genome. P. Natl. Acad. Sci. Usa. 2001; 98(20): 11479–11484. 10.1073/pnas.201394398 PMC58755 11562485

[72] Yan J, Shah T, Warburton ML, Buckler ES, McMullen MD, Crouch J. Genetic characterization and linkage disequilibrium estimation of a global maize collection using SNP markers. PloS one. 2009; 4(12): e8451 10.1371/journal.pone.0008451 20041112 PMC2795174

[73] Slatkin M. Linkage disequilibrium—understanding the evolutionary past and mapping the medical future. Nat. Rev. Genet. 2008; 9(6): 477–485. 10.1038/nrg2361 18427557 PMC5124487

[74] Zhu C, Gore M, Buckler ES, Yu J. Status and prospects of association mapping in plants. Plant Genome-US. 2008; 1(1): 5–20.

[75] Berg PR, Jentoft S, Star B, Ring KH, Knutsen H, Lien S, et al Adaptation to low salinity promotes genomic divergence in Atlantic cod (*Gadus morhua* L.). Genome Biol. Evol. 2015; 7(6): 1644–1663. 10.1093/gbe/evv093 25994933 PMC4494048

[76] Legarra A, Croiseau P, Sanchez MP, Teyssèdre S, Sallé G, Allais S, et al A comparison of methods for whole-genome QTL mapping using dense markers in four livestock species. Genet. Sel. Evol. 2015; 47(1): 6. 25885597 10.1186/s12711-015-0087-7 PMC4324410

[77] Legarra A, Ricard A, Varona L. GWAS by GBLUP: Single and Multimarker EMMAX and Bayes Factors, with an Example in Detection of a Major Gene for Horse Gait. G3-Genes Genom. Genet. 2018; 8(7): 2301–2308. 10.1534/g3.118.200336 PMC6027892 29748199

[78] Lorenz AJ, Hamblin MT, Jannink JL. Performance of single nucleotide polymorphisms versus haplotypes for genome-wide association analysis in barley. PLoS One. 2010; 5(11): e14079 10.1371/journal.pone.0014079 21124933 PMC2989918

[79] Barendse W. Haplotype analysis improved evidence for candidate genes for intramuscular fat percentage from a genome wide association study of cattle. PloS one. 2011; 6(12): e29601 10.1371/journal.pone.0029601 22216329 PMC3247274

[80] Ji-hua T, Wen-tao T, Jian-bing Y, Xi-qing M, Yi-jiang M, Jin-rui D, et al Genetic dissection of plant height by molecular markers using a population of recombinant inbred lines in maize. Euphytica. 2007; 155(1–2): 117–124.

[81] Pan Q, Xu Y, Li K, Peng Y, Zhan W, Li W, et al The genetic basis of plant architecture in 10 maize recombinant inbred line populations. Plant Physiol. 2017; 175(2): 858–873. 10.1104/pp.17.00709 28838954 PMC5619899

[82] Wang H, Liang Q, Li K, Hu X, Wu Y, Wang H, et al QTL analysis of ear leaf traits in maize (*Zea mays* L.) under different planting densities. The Crop Journal. 2017; 5(5): 387–395.

[83] Zhang J, Ku LX, Han ZP, Guo SL, Liu HJ, Zhang ZZ, et al The ZmCLA4 gene in the qLA4-1 QTL controls leaf angle in maize (Zea mays L.). J. Exp. Bot. 2014; 65(17): 5063–5076. 10.1093/jxb/eru271 24987012

[84] Zhang ZM, Zhao MJ, Ding HP, Rong TZ, Pan GT. Quantitative trait loci analysis of plant height and ear height in maize (Zea mays L.). Russ. J. Genet. 2006; 42(3): 306–310. 16649666

[85] Zhu LY, Chen JT, Li D, Zhang JH, Huang YQ, Zhao YF, et al QTL mapping for stalk related traits in maize (Zea mays L.) under different densities. J. Integr. Agr. 2013; 12(2): 218–228.

[86] Joshi AK, Chand R. Variation and inheritance of leaf angle, and its association with spot blotch (*Bipolaris sorokiniana*) severity in wheat (*Triticum aestivum*). Euphytica. 2002; 124(3): 283–291.

[87] Chen Y, Zhou Q, Tian R, Ma Z, Zhao X, Tang J, et al Proteomic analysis reveals that auxin homeostasis influences the eighth internode length heterosis in maize (*Zea mays*). Sci. Rep-UK. 2018; 8(1): 7159. 10.1038/s41598-018-23874-6 PMC5940786 29739966

